# P-226. Assessing the Risk Factors for Catheter Related Bloodstream Infections (CRBSI) among Cancer Patients with Long Term Central Venous Catheters (LCVC) for Implementing Preventive Interventions

**DOI:** 10.1093/ofid/ofae631.430

**Published:** 2025-01-29

**Authors:** Andrea Haddad, Rita Wilson Dib, Anne-Marie Chaftari, Ying Jiang, Mohammad Moussa, Hiba Dagher, Ann Philip, Ray Y Hachem, Issam I Raad

**Affiliations:** MD Anderson Cancer Center, Houston, Texas; Medical College of Georgia Medical Center, Augusta, GA; MD Anderson UT, Houston, Texas; The University of Texas MD Anderson Cancer Center, Houston, Texas; MD Anderson Cancer Center, Houston, Texas; UT MD Anderson Cancer Center, Houston, Texas; MD Anderson Cancer Center, Houston, Texas; MD Anderson UT, Houston, Texas; MD Anderson UT, Houston, Texas

## Abstract

**Background:**

Catheter Related Bloodstream Infection (CRBSI) remains a leading cause of morbidity, mortality, and escalating costs in cancer patients with LCVC despite advancements in medical care and the implementation of BSI bundle. Several risk related studies have been published in cancer patients but did not include strict definition of CRBSI and relied on Central Line-Associated Blood Stream Infections (CLABSI) definitions in the setting of Mucosal Barrier Injuries (MBI) which might be emerging from the gut rather than the catheter. Our study aims at determining specific subgroups of cancer patients who are most susceptible for CRBSIs to determine the potential effectiveness of preventive interventions such as catheter locks.

Independent predictors of CRBSI by multivariate logistic regression analysis

**Figure:**
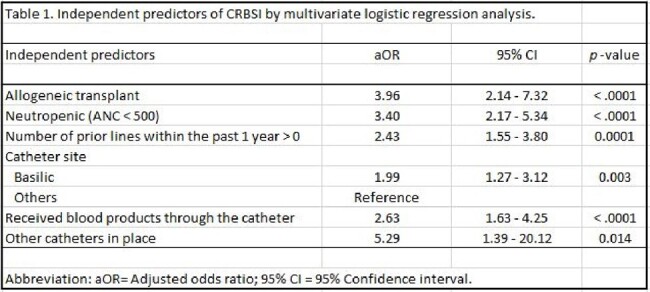

**Methods:**

We retrospectively reviewed cancer patients with indwelling LCVC that remained in place for at least 3 weeks between 2018 and 2022. By simple random sampling, we compared 200 patients who developed a definite CRBSI to 400 patients who did not. A definite CRBSI was defined according to the stringent criteria by the Infectious Diseases Society of America.

**Results:**

Among cancer patients with CRBSI, 82% had hematological malignancy. Over 50% of the catheters had 2 lumens. Patients with CRBSI were more likely to receive blood products through the catheter (81% vs 45%, p< 0.0001). The median time from catheter insertion to CRBSI was 51 days. Of the identified pathogens causing CRBSI, 62% were gram positive bacteria, 37% gram negative bacteria and 2% candida. By multivariate logistic regression analysis, the leading independent significant risk factors were the presence of multiple catheters in place, neutropenia, allogeneic transplants, followed by blood product administration, multiple catheters inserted within the past year, and basilic site insertion (table 1).

**Conclusion:**

Cancer patients with LCVC are susceptible to CRBSI, particularly those who are neutropenic, had allogeneic transplants, have multiple catheters, received blood products, or had basilic site insertion. Effective preventive interventions such as antimicrobial locks and antimicrobial catheters with prolonged activity should be primarily implemented in these highest risk populations.

**Disclosures:**

**All Authors**: No reported disclosures

